# Purified Wnt5a Protein Activates or Inhibits β-Catenin–TCF Signaling Depending on Receptor Context

**DOI:** 10.1371/journal.pbio.0040115

**Published:** 2006-04-04

**Authors:** Amanda J Mikels, Roel Nusse

**Affiliations:** **1**Department of Developmental Biology, Stanford University School of Medicine, Stanford, California, United States of America; **2**Howard Hughes Medical Institute, Stanford University School of Medicine, Stanford, California, United States of America; Cambridge UniversityUnited Kingdom

## Abstract

The Wnts comprise a large class of secreted proteins that control essential developmental processes such as embryonic patterning, cell growth, migration, and differentiation. In the most well-understood “canonical” Wnt signaling pathway, Wnt binding to Frizzled receptors induces β-catenin protein stabilization and entry into the nucleus, where it complexes with T-cell factor/lymphoid enhancer factor transcription factors to affect the transcription of target genes. In addition to the canonical pathway, evidence for several other Wnt signaling pathways has accumulated, in particular for Wnt5a, which has therefore been classified as a noncanonical Wnt family member. To study the alternative mechanisms by which Wnt proteins signal, we purified the Wnt5a protein to homogeneity. We find that purified Wnt5a inhibits Wnt3a protein–induced canonical Wnt signaling in a dose-dependent manner, not by influencing β-catenin levels but by downregulating β-catenin–induced reporter gene expression. The Wnt5a signal is mediated by the orphan tyrosine kinase Ror2, is pertussis toxin insensitive, and does not influence cellular calcium levels. We show that in addition to its inhibitory function, Wnt5a can also activate β-catenin signaling in the presence of the appropriate Frizzled receptor, Frizzled 4. Thus, this study shows for the first time that a single Wnt ligand can initiate discrete signaling pathways through the activation of two distinct receptors. Based on these and additional observations, we propose a model wherein receptor context dictates Wnt signaling output. In this model, signaling by different Wnt family members is not intrinsically regulated by the Wnt proteins themselves but by receptor availability.

## Abstract 2

Understanding signaling by Wnt proteins has been hampered by a history of conflicting data. The authors reconcile previous findings concerning Wnt signaling by using purified Wnt5a to probe the signaling pathways it activates.

## Introduction

Wnt signaling controls a variety of adult and developmental processes, largely by modulating gene transcription [ 1]. The necessity of precise regulation to prevent the inappropriate activation of Wnt signaling is underscored by the fact that misregulation of several components of the canonical Wnt signal transduction pathway leads to tumorigenesis [ 2]. Wnt signaling is also thought to play a key role in controlling stem cell fate [ 3]. Thus, understanding the mechanisms that regulate Wnt signaling is of critical importance.

Wnt proteins are found in all metazoan organisms and as many as 19 mammalian homologs are known (Wnt home page: http://www.stanford.edu/~rnusse/wntwindow.html). While homologs have a high degree of sequence similarity, expression of different Wnt proteins can lead to vastly different developmental outcomes. In the most well-understood “canonical” Wnt/β-catenin signaling pathway, in the absence of a Wnt ligand, the main mediator of the signal relay, β-catenin, is bound in a cytosolic protein complex containing Axin, the adenomatous polyposis coli gene product (APC), glycogen synthase kinase-3β (GSK-3β), and other proteins. Axin and APC serve as scaffolding proteins that enable GSK-3β to phosphorylate β-catenin, thereby targeting it for ubiquitination by βTrCP (beta-transducin repeat–containing homologue protein) and subsequent degradation in the proteasome. Cytosolic β-catenin protein levels are thus kept low in the absence of ligand stimulation. Wnt protein binding to cognate Frizzled (Fz) and low-density lipoprotein (LDL) receptor–related protein (LRP)5/6 coreceptors leads to the activation of the Dishevelled (Dvl) protein, which then inhibits GSK-3β–mediated phosphorylation of β-catenin. Cytosolic β-catenin protein becomes stabilized and newly synthesized β-catenin is able to accumulate and then translocate to the nucleus where binding to T-cell factor (TCF)/lymphoid enhancer factor (LEF) transcription factors leads to the activation of target gene expression [ 1].

Prior to the discovery of the Fz and LRP coreceptors, Wnt proteins were classified into two functional groups based on the observation that ectopic expression of some Wnts, such as Wnt1 and Wnt3a, is sufficient to induce a secondary dorsal-ventral axis in *Xenopus* embryos and morphologically transform C57MG mouse mammary epithelial cells, whereas expression of “Wnt5a class” Wnts, including Wnts 4, 5a, and 11, is not sufficient [ 4– 8]. One hypothesis for how the structurally similar, although functionally distinct, extracellular Wnt ligands trigger different developmental outcomes is that the two classes of Wnts signal via different intracellular pathways. Indeed, experiments in zebrafish and *Xenopus* embryos using mRNA injection to activate Wnt signaling have suggested that expression of Wnt5a stimulates intracellular calcium (Ca ^2+^) flux leading to the activation of Ca ^2+^–dependent effector molecules such as calcium/calmodulin–dependent kinase II (CamKII), nuclear factor associated with T cells (NFAT), and protein kinase C (PKC) in a pertussis toxin (PTX)–sensitive manner [ 9– 12]. However, due to the lack of active soluble Wnt5a protein, direct activation of “Wnt/Ca ^2+^” pathway signaling in mammalian cell culture systems has not been fully investigated.

In addition to activating alternative signaling pathways, Wnt5a may also inhibit Wnt/β-catenin signaling. Early experiments in *Xenopus* embryos showed that coexpression of *X*Wnt5a with *X*Wnt8 abrogates the ability of *X*Wnt8 to induce a secondary axis [ 13– 15]. Wnt5a knockout mice show increased β-catenin signaling in the distal limb, indicating that Wnt5a may inhibit β-catenin stabilization [ 16]. In contrast, experiments from Ishitani et al. have shown that Wnt5a-induced Ca ^2+^ flux blocks canonical signaling downstream of β-catenin stabilization by inhibiting TCF-mediated transcription in a PTX-sensitive manner [ 16– 18]. As Wnt5a heterozygous mice develop myeloid leukemias and B-cell lymphomas, an intriguing hypothesis is that Wnt5a serves as a tumor suppressor in part by preventing excess Wnt/β-catenin signaling [ 19].

While most Wnt signaling has been attributed to the activation of Fz receptors, an alternative possibility is that Wnts carry out their diverse roles by signaling via different receptors. One such receptor is Ror2, an orphan tyrosine kinase possessing an extracellular cysteine-rich Wnt binding domain (CRD) [ 20– 23]. Wnt5a and mRor2 have overlapping expression patterns, their knockout phenotypes are similar, and recently mRor2 has been shown to act synergistically with Wnt5a to activate Jun kinase [ 23– 26]. Thus, Wnt5a may be mediating its inhibitory role through the activation of this novel receptor.

There are conflicting data in the literature regarding the mechanism by which Wnt5a and other so-called noncanonical Wnt protein signals. To understand how signals like Wnts influence cells, one must discriminate between early and late effects and measure those effects in a concentration-dependent manner. To that end, we have purified active Wnt5a protein and established quantitative assays for signaling. We show for the first time that soluble Wnt5a protein is able to directly inhibit canonical Wnt signaling. Treatment of cells with purified protein over short time intervals is sufficient to inhibit the activation of the TCF/LEF-driven luciferase reporter SuperTopFlash (STF). The inhibition observed is dose responsive, occurs downstream of β-catenin stabilization, and is PTX insensitive. We also show that an alternative Wnt receptor, Ror2, is required for Wnt5a-mediated inhibition of canonical signaling and that the extracellular, cysteine-rich Wnt binding domain and intracellular, cytoplasmic domain are crucial for the receptor's inhibitory function. Whereas Wnt5a protein is usually associated with noncanonical Wnt signaling, we show that Wnt5a can activate Wnt/β-catenin signaling in the presence of Fz 4 and LRP5. Based on these and other observations, we propose a model wherein receptor context dictates Wnt signaling output, suggesting a new layer of regulatory complexity in the Wnt signaling cascade with important implications in development and disease.

## Results

### Purification of Wnt5a Protein: Evidence that It Is Cleaved from a Precursor

**Figure 1:**
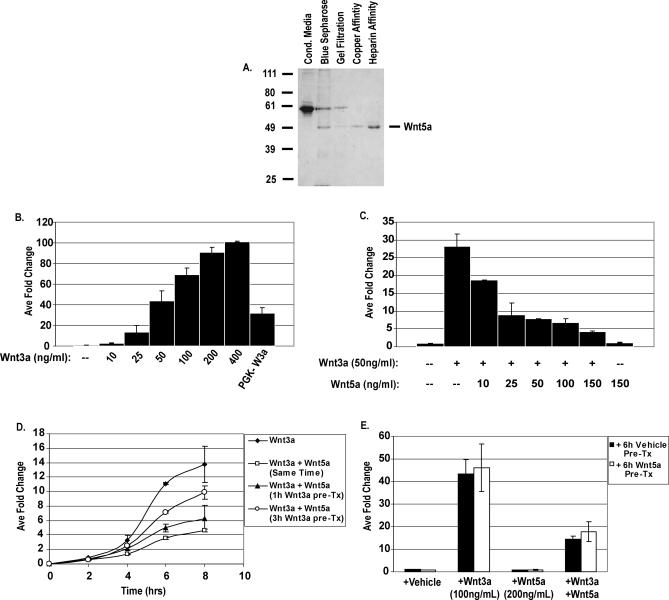
Purified Wnt5a Protein Inhibits Canonical Wnt Reporter Activation (A) Coomassie blue–stained gel showing the Wnt5a protein purification scheme. (B) The 24-h Wnt3a protein treatment activates the STF luciferase reporter in a dose-responsive manner. (C) Concomitant Wnt5a protein treatment inhibits Wnt3a-induced STF reporter activation. (D) Time-course analysis of Wnt5a-mediated inhibition of Wnt3a. At approximately 40 h post-transfection, cells were treated with Wnt3a protein for 0, 2, 4, 6, or 8 h alone or in conjunction with Wnt5a at *t* = 0, *t* = 1 h, or *t* = 3 h. (Filled diamonds indicate Wnt3a treatment alone; open diamonds indicate Wnt3a treatment at *t* = 0 plus Wnt5a protein treatment at *t* = 0; filled triangles indicate Wnt3a treatment at *t* = 0 plus Wnt5a protein treatment at *t* = 1; open circles indicate Wnt3a treatment at *t* = 0 plus Wnt5a protein treatment at *t* = 3.) (E) Wnt5a pretreatment does not enhance its inhibitory activity. At 24 h post-transfection, cells were pretreated with vehicle or Wnt5a protein (200 ng/ml) for 8 h. Cells were then treated with vehicle, Wnt3a alone, or Wnt3a concomitantly with Wnt5a for an additional 24 h, and luciferase assay was performed. (Filled squares indicate cells that were pretreated with vehicle for 8 h prior to Wnt addition; open squares indicate cells pretreated with Wnt5a protein for 8 h prior to Wnt addition.) Unless stated in text, luciferase activity was measured 48 h after transfection, approximately 24 h after Wnt treatment, and results are shown as the average fold change ± SD in luciferase activity over transfected, untreated negative controls.

We purified the Wnt5a protein from cells overexpressing the mouse *Wnt5a* gene using methods derived from those developed for other members of the Wnt family with several modifications (Figure1A) [ 27]. Throughout the purification, we followed the Wnt5a protein using an antibody that detects Wnt5a on a Western blot. Later steps in the purification were also monitored by activity assays (see below). To unambiguously identify the purified protein as the product of the *Wnt5a* gene, we determined the amino-terminal sequence. We found that the mature protein starts with a sequence IIGAQPLCSQLAGLSQGQKKL, a sequence beginning 62 amino acids downstream from the predicted initiator methionine of *Wnt5a* and 24 amino acids from the predicted signal cleavage site between amino acids 37 and 38 [ 28]. While all Wnt proteins contain signal sequences required for secretion from cells, generally they are not internally cleaved at a site downstream from the signal sequence. The exception, interestingly, is the *Drosophila* ortholog of Wnt5a; DWnt5 protein is made as a precursor of more than 1,000 amino acids weighing 140 kDa and cleaved into a smaller mature protein of 80 kDa [ 29]. It is not known whether the processing of the Wnt5a molecule involves specific proteases.

We find that, similar to other Wnt proteins that we have purified, the Wnt5a protein is hydrophobic as it requires detergent to stay in solution and partitions in a detergent phase when subjected to extraction (unpublished data) [ 27]. We presume that the protein, like Wnt3a, is modified by the covalent attachment of a palmitate, but we have not examined this further. Significantly, the cysteine residue that is modified by palmitoylation in Wnt3a is conserved in Wnt5a, even after the internal cleavage of the protein.

### Purified Wnt5a Protein Inhibits Canonical Wnt Reporter Activation

**Figure 2:**
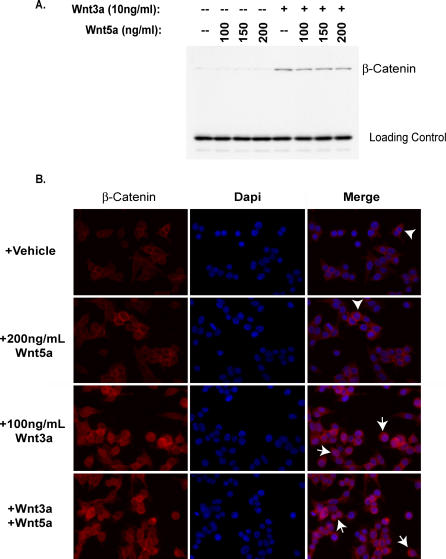
Wnt5a Does Not Affect β-Catenin Stabilization (A) Wnt5a protein treatment does not affect cytosolic β-catenin protein levels. The 293 cells were treated for 3 h with the indicated concentrations of Wnt proteins and then hypotonically lysed for Western blot analysis of cytosolic fractions. (B) Wnt5a protein treatment does not alter β-catenin cellular localization. Cells grown on coverslips were treated with the indicated concentrations of Wnt proteins for 6 h and then fixed and stained as described in the text. Membrane localized β-catenin is indicated by arrowheads; membrane and cytosolic staining is indicated by arrows.

Standard readouts for the activity of various Wnt family members include an increase in the level of the β-catenin protein and the activation of reporter genes, in particular, the Topflash reporter that contains TCF binding sites upstream of a luciferase transgene. We found that when transiently transfected into 293 cells, the SuperTopflash (STF) luciferase reporter variant [ 30] is robustly activated in response to Wnt3a treatment and exhibits dose-dependency. As shown in Figure1B, Wnt3a protein treatment induces a 100-fold increase in reporter activation and a linear dose response when the concentration is lowered. In accordance with other groups, we observe that Wnt5a protein by itself does not lead to the activation of the STF reporter in 293 cells, nor does it alter levels of β-catenin protein ( Figures 1C and 2A) [ 16, 31]. Based on reports from Ishitani et al. and others [ 14, 17, 32, 33], we then tested whether the addition of Wnt5a protein could inhibit Wnt3a-induced reporter activity. Using a concentration of Wnt3a protein within the linear range of reporter activation (50 ng/ml), Wnt5a protein was added to cells concomitantly with Wnt3a. As shown in Figure 1C, Wnt5a elicits a dose-responsive decrease in Wnt3a-mediated reporter activation.

Wnt5a protein inhibits Wnt3a signaling at the earliest time points following reporter activation. When cells are pretreated with Wnt3a protein for 1 or 3 h prior to Wnt5a treatment, Wnt5a can inhibit Wnt3a-induced reporter activation within 1 h of exposure ( Figure 1D). In addition, Wnt5a pretreatment of cells for 8 h followed by washing has no effect on either Wnt3a-induced STF reporter activation or Wnt5a-mediated inhibition, suggesting that Wnt5a does not induce the accumulation of an inhibitory factor ( Figure 1E). These data taken together indicate that purified Wnt5a protein is sufficient to rapidly inhibit canonical Wnt signaling in a potentially post-translational fashion.

### Wnt5a Does Not Affect β-Catenin Protein Stabilization

One possible explanation for the observed abrogation of reporter activation is that Wnt5a protein competes with Wnt3a for Fz receptor binding sites. Alternatively, Wnt5a has been proposed to inhibit canonical Wnt signaling via upregulation of *Siah-2,* which targets β-catenin for βTrCP-independent proteasomal degradation [ 16]. In both of these models, β-catenin protein levels should be reduced following concomitant Wnt5a treatment compared to Wnt3a protein treatment alone; in the former case, signal transduction is blocked at the level of the receptor, whereas in the latter, any β-catenin protein stabilized due to Wnt3a signal activation should be appreciably degraded by the opposing effects of Wnt5a.

To assess the levels of accumulated β-catenin protein, we prepared cytosolic extracts from 293 cells treated for 3 h with Wnt3a protein, Wnt5a protein, or both. Cellular fractionation is necessary to ensure that only cytosolic β-catenin protein stabilized due to active Wnt signaling is observed as opposed to the relatively stable membrane-associated pool of β-catenin protein. Although a particularly low dose of Wnt3a protein (10 ng/ml) was used in this analysis to ensure that subtle differences in β-catenin protein levels could be discerned, no appreciable reduction in β-catenin accumulation was observed ( Figure 2A), even when concentrations of Wnt5a protein (200 ng/ml) sufficient to inhibit the STF reporter 5-fold were used. These data show that the Wnt3a signal is initiated in the presence of Wnt5a protein, indicating that Wnt5a does not efficiently compete with Wnt3a for receptor binding sites.

To determine whether Wnt5a treatment inhibits β-catenin nuclear entry, cell-staining experiments were performed ( Figure 2B). The 293 cells treated with vehicle or Wnt5a protein alone display a membrane-associated staining pattern (arrowheads) consistent with β-catenin's role in cell adhesion. By contrast, cells treated with Wnt3a protein alone appeared brighter than vehicle-treated cells, with β-catenin observed in the cytoplasm and nuclei (arrows). No significant difference in β-catenin signal intensity or localization was observed when cells were treated with Wnt3a in combination with Wnt5a protein ( Figure 2B). The observation that Wnt3a-mediated β-catenin stabilization and nuclear entry appear unaffected by Wnt5a treatment suggests that upregulation of *Siah-2,* and subsequent degradation of β-catenin, is not the primary mechanism of Wnt5a protein-mediated inhibition of gene transcription in these cells.

### Wnt5a-Mediated Inhibition of Canonical Signaling Is Not Associated with Wnt-Stimulated Ca ^2+^ Flux

Past studies in zebrafish and *Xenopus* embryos have suggested that overexpression of Wnt5a can trigger intracellular Ca ^2+^ flux, leading to the activation of Ca ^2+^-dependent effector molecules such as CamKII [ 9, 34, 35]. It has been proposed that active CamKII protein can then initiate the mammalian TGF-β–activated kinase 1(TAK)/Nemo-like kinase (NLK) mitogen-activated protein kinase signaling cascade, resulting in NLK-mediated phosphorylation of TCF/LEF transcription factors. This phosphorylation of TCF/LEF prevents the β-catenin–TCF/LEF transcriptional complex from binding to DNA [ 17, 18, 36]. In this model, inhibition of Wnt/β-catenin signaling due to Wnt5a-stimulated Ca ^2+^ flux occurs downstream of β-catenin stabilization and at the level of TCF-mediated transcription. In a variety of organisms and assays, Ca ^2+^ signaling induced by Wnts has consistently been shown to be PTX *sensitive,* as the Fz receptor described as transmitting the inhibitory Wnt signal (RFz2) is thought to be coupled to a PTX-sensitive G protein [ 8, 37, 38].

**Figure 3:**
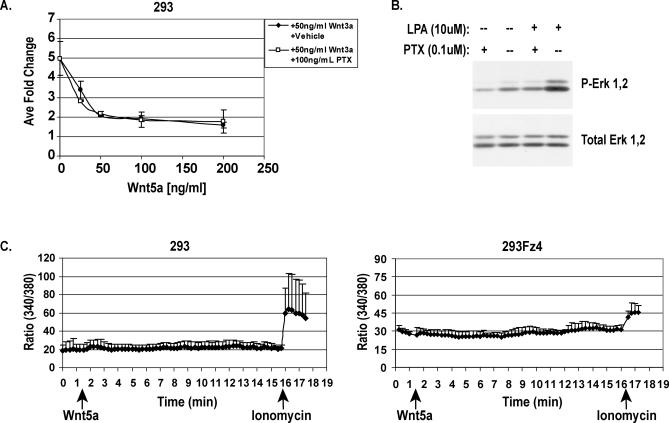
Wnt5a-Mediated Inhibition of Canonical Signaling Is Not Associated with Wnt-Stimulated Ca ^2+^ Flux (A) Wnt5a-mediated inhibition is PTX insensitive. At 24 h post-transfection, 293 cells were pretreated with vehicle or 100 ng/ml PTX for 24 h. Cells were then treated with indicated Wnt proteins concomitantly with vehicle or PTX for an additional 24 h, and luciferase assay was performed. (B) PTX is active in 293 cells as PTX pretreatment of cells inhibits LPA-induced Erk1,2 activation. The 293 cells were pretreated with 100 ng/ml PTX for 24 h and then treated with 10 μM LPA or vehicle for 10 min. Western blot analysis was then performed on total cell lysates using anti–phospho-Erk1,2 antibody. Membrane was stripped and reprobed for total Erk1,2 as a loading control. (C) Wnt5a protein does not directly stimulate intracellular Ca ^2+^ flux. The 293 and 293Fz4 cells were loaded with Fura-2-dextran and then monitored for changes in intracellular Ca ^2+^ concentration as determined by the change in 340/380 excitation wavelength ratio following Wnt5a (500 ng/ml) and subsequent ionomycin treatment. Data represent the average 340/380 excitation wavelength ratio of three or more independent cells within the same field ± SD. Wnt5a has little effect on the ratio over the 15-min time course, whereas ionomycin treatment rapidly and robustly induces Ca ^2+^ flux in these cells. Prolonged treatment with Wnt5a (up to 1 h) did not result in changes in intracellular Ca ^2+^ levels (unpublished data).

To determine whether Wnt5a protein inhibits β-catenin signaling via the direct activation of intracellular Ca ^2+^ flux downstream of heterotrimeric G proteins, 293 cells transiently transfected with the STF reporter were treated with 50 ng/ml W3a protein and increasing doses of Wnt5a protein following 24-h pretreatment with vehicle or 100 ng/ml (0.1 μM) PTX. Figure 3A shows that Wnt5a protein-mediated inhibition of the STF reporter was not affected by PTX treatment. The PTX used was active in these cells, as lysophosphatidic acid (LPA) treatment of cells following pretreatment with PTX was unable to stimulate the phosphorylation of the mitogen-activated protein kinases Erk1 and Erk2, a known PTX-sensitive process in 293 cells [ 39] ( Figure 3B). These data are consistent with Topol et al.'s [ 16] findings that overexpression of a dominant negative version of CamKII, and treatment of cells with a specific inhibitor of CamKII, does not perturb Wnt5a-mediated inhibition of Wnt/β-catenin signaling in 293 cells.

Wnt5a protein was also assayed for its ability to directly stimulate intracellular calcium flux. Figure 3C shows that Wnt5a protein treatment at high doses does not alter the intracellular concentration of Ca ^2+^ in 293 cells, although subsequent ionomycin treatment of the same cells promotes robust Ca ^2+^ flux. Similar results were observed in 293 cells stably expressing mouse Fz4 (mFz4); a Wnt receptor thought to be involved in Wnt-stimulated calcium flux ( Figure 3C) [ 40]. In addition, transient treatment with Wnt5a protein does not activate other Ca ^2+^-sensitive reporter constructs such as an NFAT-responsive luciferase reporter (unpublished data). These data taken together strongly suggest that Wnt-stimulated Ca ^2+^ flux is not the direct mechanism utilized by Wnt5a to inhibit canonical Wnt signaling.

### Wnt5a Protein Activates β-Catenin Signaling Depending on Receptor Context

**Figure 4:**
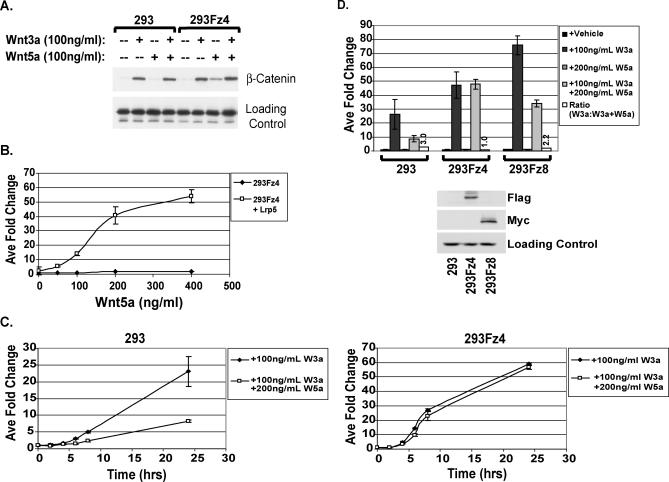
Wnt5a Protein Activates β-Catenin Signaling Depending on Receptor Context (A) Wnt5a treatment leads to β-catenin stabilization specifically in cells expressing mFz4. The 293 and 293Fz4 cells were treated with Wnt proteins and then assayed for cytosolic β-catenin protein accumulation via Western blot analysis. (B) Wnt5a treatment activates the STF reporter when LRP5 is coexpressed. At 24 h post-transfection with STF reporter and LRP5 or empty vector, 293Fz4 cells were treated with increasing concentrations of Wnt5a protein for an additional 24 h, and luciferase assay was performed as described in Figure 1. (C) Wnt5a does not inhibit Wnt3a-mediated reporter activation in 293Fz4 cells. The 293 and 293Fz4 cells were treated with Wnts as indicated 24 h post-transfection with reporters, and luciferase assay was performed. (D) Loss of Wnt5a-mediated reporter inhibition is specific to mFz4 overexpression. The 293, 293Fz4, and 293Fz8 cells were treated with the indicated Wnts for 24 h, 24 h post-transfection, and then luciferase assay was performed. Wnt5a maintains inhibitory activity when mFz8, but not mFz4, is overexpressed.

Although we show that Wnt5a protein can inhibit canonical Wnt signaling, the question remains of whether Wnt5a can *activate* Wnt/β-catenin signaling in mammalian systems. Multiple Fz receptors, including mFz4, mFz6, mFz7, and mFz8, were assayed for their ability to transduce Wnt5a-mediated canonical Wnt signaling (unpublished data). We found that Wnt5a could indeed activate Wnt/β-catenin signaling when cells overexpressed mFz4; none of the other overexpressed receptors could transduce the Wnt5a signal. Western blot analysis of cytosolic extracts probed for β-catenin ( Figure 4A) shows that while Wnt5a has no effect on cytosolic β-catenin levels in the parental 293 cell line, Wnt5a can induce modest β-catenin accumulation in cells stably expressing a FLAG-tagged mFz4 construct (293Fz4). These results are in accordance with observations by Umbhauer et al. [ 41] that coinjection of *X*wnt5a RNA with *X*fz4 RNA leads to the synergistic activation of Wnt/β-catenin target genes in *Xenopus* animal caps.

We next tested whether Wnt5a could activate the STF reporter in 293Fz4 cells. Neither Wnt5a protein treatment at high doses nor transfection of Wnt5a DNA into cells expressing mFz4 could activate the STF reporter. However, as shown in Figure 4B, when LRP5 is coexpressed with mFz4, Wnt5a protein is able to activate the luciferase reporter. Thus, Wnt5a can activate TCF/β-catenin signaling given the expression of the appropriate receptors. Interestingly, Wnt5a is unable to activate the STF reporter in 293Fz4 cells transiently transfected with LRP6, indicating the specificity of the Wnt5a/Fz4/LRP5 signaling complex (unpublished data).

To determine whether Wnt5a protein can inhibit Wnt3a-mediated STF reporter activation in 293Fz4 cells, 293 and 293Fz4 cells were transiently transfected with the STF reporter and then treated with Wnt proteins over the course of 24 h. Whereas Wnt5a protein potently inhibits the STF reporter in parental 293 cells, Wnt5a no longer inhibits Wnt3a-induced reporter activation in 293Fz4 cells ( Figure 4C). This effect was specific to mFz4, as stable expression of mFz8 was not sufficient to abrogate Wnt5a-mediated inhibition of STF reporter activation ( Figure 4D). Additionally, Wnt5a does not stabilize β-catenin protein in the presence of mFz8 (unpublished data). Thus, specifically in the context of mFz4 and LRP5 expression, Wnt5a is unable to inhibit Wnt3a-mediated β-catenin signaling and instead induces β-catenin accumulation and STF reporter activation.

### An Alternative Wnt Receptor, mRor2, Mediates Wnt5a's Inhibitory Activity

One explanation for why Wnt5a is unable to inhibit Wnt3a-mediated β-catenin signaling when mFz4 is overexpressed is that in 293Fz4 cells, increased mFz4 receptor binding sites may titrate available Wnt5a protein away from an alternative receptor carrying out its inhibitory function. To investigate this hypothesis, we turned to the orphan receptor mRor2, a single-pass transmembrane tyrosine kinase with proposed kinase-dependent and -independent activities [ 25, 42]. mRor2 is an appealing candidate receptor mediating Wnt5a's inhibitory activity because Wnt5a and mRor2 have overlapping expression patterns, their knockout phenotypes are similar, and mRor2 has been shown previously to act synergistically with Wnt5a to activate Jun kinase [ 22– 26]. In addition, CAM-1, the C. elegans Ror2 homolog, has been shown to inhibit canonical Wnt signaling–mediated cell migration, although the Wnt ligand mediating this inhibition remains to be determined [ 21].

**Figure 5:**
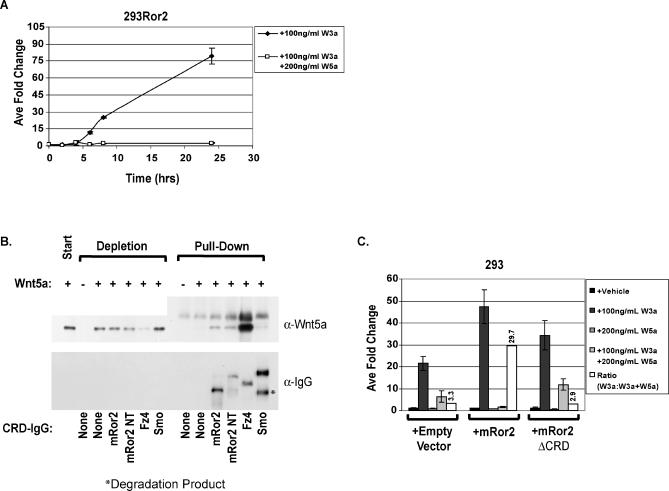
An Alternative Wnt Receptor, mRor2, Mediates Wnt5a's Inhibitory Activity (A) Wnt5a synergizes with mRor2 to inhibit Wnt3a-mediated reporter activation. The 293 cells stably expressing exogenous mRor2 (293Ror2) were treated with Wnts as indicated, 24 h post-transfection with reporters, and luciferase assay was performed as outlined in Figure 1. (B) Wnt5a binds directly to the Fz4 and mRor2 CRD domains. Wnt5a protein specifically binds to the purified mFz4 and mRor2 CRDs; only background binding to the Smoothened CRD negative control is observed. While it may appear that Wnt5a binds to a similar extent to the Ror2 CRDs and Smo CRD, the input levels of the Smo CRD-IgG exceeded the Ror2 CRD-IgG levels, accounting for the increased background binding Smo CRD-IgG. (C) The mRor2 CRD domain is required for mediating Wnt5a inhibitory activity. The 293 cells were transiently transfected with reporters along with empty vector, mRor2, or mRor2ΔCRD constructs, treated with Wnts as described in text 24 h post-transfection for an additional 24 h, and then luciferase assay was performed as outlined in Figure 1.

To determine whether Wnt5a mediates its inhibitory effects through the mRor2 receptor, 293 cells stably expressing the full-length mRor2 receptor (293mRor2) were treated with Wnt proteins over the course of 24 h. As shown in Figure 5A, Wnt3a protein treatment robustly activates the STF reporter in the presence of mRor2. However, at every time point measured, Wnt5a protein-mediated inhibition of Wnt/β-catenin signaling is synergistically enhanced by mRor2 overexpression as compared to the parental cell line (compare to Figure 4C and see Figure S1).

We next assayed whether mRor2 was carrying out its inhibitory function through direct binding to Wnt5a. Two variants of the extracellular cysteine-rich Wnt binding domain (CRD) of mRor2 were cloned in frame amino-terminal to an immunoglobulin heavy chain domain (IgG) as previously described and expressed in 293 cells [ 43]. One variant is comprised of the Ror2 CRD alone cloned downstream of an exogenous signal sequence (Ror2 CRD-IgG); the other variant utilizes the endogenous Ror2 signal sequence and possesses the full amino terminus of the Ror2 protein ending at the carboxyl terminus of the CRD (Ror2 NT CRD-IgG). The secreted mRor2 CRD-IgG fusion proteins were then purified from conditioned media, bound to protein A beads, and tested for their ability to bind to purified Wnt proteins. The Smoothened receptor is a Fz family member that possesses a CRD domain but, rather than playing a role in Wnt signaling, it functions exclusively Hedgehog signal transmission. As a negative control, purified Smoothened (Smo) CRD-IgG protein was also bound to beads and assayed for Wnt binding. Figure 5B shows that Wnt5a protein specifically binds to the purified Ror2 and Fz4 CRD domains, whereas only background binding to the Smoothened CRD occurs. Wnt3a protein shows specific binding only to the Fz4 CRD-IgG protein (unpublished data).

We next utilized a Ror2 deletion construct that lacks the extracellular cysteine-rich Wnt-binding domain (mRor2ΔCRD) to determine whether the CRD domain was necessary for Wnt5a signal transmission. When transiently transfected into 293 cells in conjunction with the STF reporter, mRor2ΔCRD does not enhance Wnt5a-mediated inhibition of the STF reporter ( Figure 5C). Thus, the CRD domain binds directly to Wnt5a and is required for Ror2 to transduce Wnt5a's inhibitory activity.

### Expression of mRor2 Is Required for Wnt5a-Mediated Inhibition of Canonical Signaling

**Figure 6:**
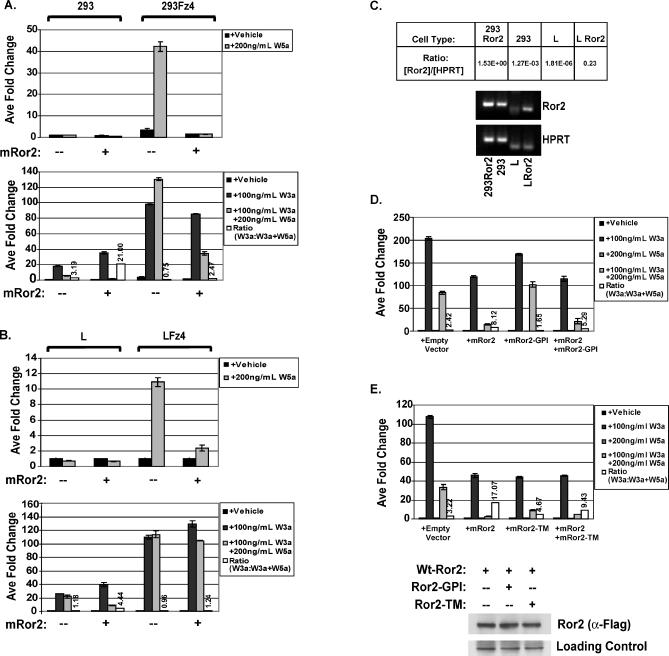
Expression of mRor2 Is Required for Wnt5a-Mediated Inhibition of Canonical Signaling (A) In 293 cells, mRor2 overexpression inhibits Wnt5a-mediated activation of the STF reporter. The 293 and 293Fz4 cells were transiently transfected with the reporters and LRP5 with or without mRor2 and then treated with Wnt proteins for 24 h, 24 h post-transfection. Overexpression of wild-type mRor2 inhibits Wnt5a-mediated activation of the STF reporter as well as allowing Wnt5a to inhibit Wnt3a-mediated STF reporter activation in 293Fz4 cells. (B) mRor2 overexpression inhibits Wnt5a-mediated activation of the STF reporter and allows Wnt5a to block Wnt3a-induced STF reporter activation in mouse L cells. L and LFz4 cells were transiently transfected with the reporters and LRP5 with or without mRor2 and then treated with Wnt proteins for 24 h, 24 h post-transfection. (C) Quantitative real-time PCR analysis of Ror2 expression in 293 and L cells. Reverse transcription followed by 45 cycles of quantitative real-time amplification of 293 cell RNA reveals a robust Ror2 product that is over 700 times more abundant than that produced from RNA derived from mouse L cells. Hprt quantification serves as an internal normalization control and RNA derived from 293Ror2 and LRor2 cells serves as a positive control for the reaction. The products from the PCR analysis are displayed below the charted quantitative data. (D) The cytoplasmic domain of mRor2 is required for its inhibitory function. Expression of a membrane-tethered variant of mRor2 possessing the extracellular domain of mRor2 fused to a GPI linkage (mRor2-GPI) reduces Wnt5a's ability to inhibit Wnt3a induced canonical Wnt signaling as well as inhibits wild type mRor2′s ability to enhance Wnt5a-mediated inhibition. (E) Expression of a membrane-tethered variant of mRor2 (mRor2-TM) that contains the extracellular and transmembrane domains of mRor2, but lacks the cytoplasmic domain, does not enhance Wnt5a mediated inhibition of canonical Wnt signaling. Expression of mRor2-TM reduces Wnt5a's ability to inhibit Wnt3a-induced canonical Wnt signaling when wild-type mRor2 is overexpressed. Western blot analysis shows that wild-type mRor2 levels do not change when the truncated mRor2 variants are coexpressed.

We next tested whether increased mRor2 expression was necessary to shift the balance of Wnt5a-mediated activation versus inhibition of Wnt/β-catenin signaling in cells overexpressing mFz4. 293 and 293Fz4 cells were transiently transfected with the STF reporter and mRor2 and then treated with the indicated Wnt proteins. Figure 6A shows that Wnt5a-induced reporter activation in the presence of mFz4 and LRP5 is abrogated when mRor2 is expressed. In addition, while Wnt5a protein is unable to inhibit Wnt/β-catenin signaling in the presence of mFz4, as compared to parental 293 cells, mRor2 overexpression synergistically enhances Wnt5a's ability to inhibit the reporter gene in both cell lines. Once again, inhibition of Wnt3a-mediated reporter activation was observed only in the presence of Wnt5a; mRor2 expression alone did not reduce Wnt3a-mediated reporter activation, indicating that the inhibitory activity is dependent on the presence of Wnt5a.

Wnt5a's inhibitory activity was also tested in another cell line, mouse L cells. Whereas Wnt5a protein treatment in parental 293 cells robustly inhibits the Wnt3a-mediated activation of the STF reporter, Wnt5a treatment has little to no inhibitory affect in L cells ( Figure 6B). To assess whether this is due to reduced expression of mRor2, L cells were transfected with mRor2 and then treated with Wnt proteins. As shown in Figure 6B, when mRor2 is overexpressed, Wnt5a protein potently inhibits the Wnt3a-induced reporter activation in L cells. Again, mRor2 expression has no inhibitory effect on Wnt3a-stimulated reporter activation in the absence of Wnt5a. Although Wnt5a is able to activate the luciferase reporter in L cells stably expressing mFz4 and transiently transfected with LRP5, mRor2 cotransfection effectively inhibits Wnt5a's canonical signaling ability ( Figure 6B).

Quantitative RT-PCR analysis was performed to ascertain the expression of Ror2 in 293 and L cells. A specific Ror2 transcript in the L cell sample did not emerge until after 40 cycles of amplification, indicating that Ror2 is expressed in almost immeasurably low abundance in L cells. Quantification of the real-time data revealed that Ror2 transcripts were over 700 times more abundant in 293 cells than in L cells ( Figure 6C). mRor2 expression thus appears to be required for Wnt5a-mediated inhibition of Wnt/β-catenin signaling in mouse L cells.

It has been proposed that the C. elegans Ror protein CAM-1 has tyrosine kinase–independent functions [ 42, 44] and that *Xenopus X*Ror2 can function in the absence of its cytoplasmic domain [ 45]. We thus created a membrane-tethered variant of mRor2 (mRor2-GPI) to assay whether mRor2 could still function in the absence of its transmembrane and cytoplasmic domains. Expression of mRor2-GPI reduced Wnt5a's ability to inhibit Wnt3a-induced STF reporter activation when transfected into 293 cells as compared to cells transfected with the reporters alone ( Figure 6D). In addition, when Ror2-GPI was transfected into cells in conjunction with wild-type mRor2, mRor2-GPI blocked wild-type mRor2′s enhancement of Wnt5a-mediated inhibition, suggesting that the membrane-tethered variant of mRor2 serves as a dominant negative. The fact that expression of mRor2-GPI alone did not enhance Wnt5a-mediated inhibition indicates that the cytoplasmic, and potentially the transmembrane, domain is required for mRor2 to carry out its inhibitory function.

To assess the role the mRor2 transmembrane domain plays in mediating the Wnt5a signal, we created a mRor2 construct that contains the endogenous extracellular and transmembrane domains of the protein but lacks the cytoplasmic domain (Ror2-TM). We find that similar to the mRor2-GPI construct, expression of Ror2-TM was sufficient to inhibit wild-type mRor2′s ability to enhance Wnt5a signaling ( Figure 6E). Unlike the mRor2-GPI construct, however, the mRor2-TM construct did not reduce Wnt5a's ability to inhibit Wnt3a-induced STF reporter activation when transfected into 293 cells alone. Western blot analysis indicates that wild-type mRor2 expression is unaffected by cotransfection with mRor2-GPI or mRor2-TM, further strengthening the idea that these constructs serve as dominant negative variants of mRor2. These data taken together suggest that mRor2 does not simply present Wnt5a to another receptor but rather that Wnt5a binding initiates Ror2-induced cytoplasmic signaling events.

## Discussion

Due to early overexpression studies in *Xenopus* embryos, the Wnts were grouped into various classes based on their canonical Wnt signaling ability without regard for the cellular context in which they were overexpressed. Receptor expression, however, clearly plays a role in determining signaling output; when coexpressed with the appropriate Fz receptor, the prototypical “noncanonical” Wnt, *X*Wnt5a, can signal in a canonical fashion to induce the formation of a secondary axis [ 46]. Whereas some have argued that the lack of functional interaction with LRP distinguishes Wnt5a from so-called canonical Wnts in mammalian cells [ 31], previous studies and our present work confirm that Wnt signaling output is not intrinsically related to the Wnt protein itself but rather due to a combination of factors including receptor availability [ 41, 46].

Several controversies exist in the literature today regarding the mechanisms by which Wnt proteins signal. Much of the conflicting data, however, can be attributed to the variety of cellular and organismal systems studied coupled with the previous lack of soluble, active Wnt proteins. In the case of Wnt5a, for example, one report has shown that overexpression of Wnt5a inhibits canonical signaling by promoting the degradation of β-catenin protein [ 16]. By contrast, we found that, in accordance with other groups, Wnt5a protein treatment has no affect on β-catenin protein levels but rather inhibits canonical Wnt signaling at the level of TCF transcription [ 17, 18, 33]. Although our data are in agreement with Wnt5a overexpression studies by Ishitani et al. [ 17] with respect to the inhibition occurring downstream of β-catenin protein stabilization, we found that Wnt5a does not stimulate Ca ^2+^ flux and that Wnt5a-mediated inhibition is not sensitive to pertussis toxin treatment. These data effectively eliminate G protein–mediated activation of calcium signaling as the primary mechanism of Wnt5a-mediated inhibition.

Fz receptor signaling capabilities have also often been debated. Previous studies pertaining to Fz4′s role in the disease familial exudative vitreoretinopathy have suggested that the Fz4 receptor does not mediate Wnt/β-catenin signaling but rather elicits intracellular calcium flux that subsequently activates downstream calcium effector molecules [ 34, 40]. By contrast, we show here that in multiple cell lines, Fz4 allows Wnt5a to specifically *activate* TCF/Lef transcription in the presence of LRP5 and that mFz4 expression does not enhance calcium flux ( Figure 3C). The true signaling capabilities of the Fz4 receptor may have been previously overlooked due to the use of an inappropriate Wnt ligand in the analysis, Wnt1 as opposed to Wnt5a. Additionally, as calcium signaling capabilities were attributed to Fz4 in the absence of exogenous ligand stimulation, it remains to be seen whether Wnt ligands are required for Fz-mediated Ca ^2+^ flux.

We observe that when mFz4 is overexpressed alone, Wnt5a protein treatment is sufficient to induce β-catenin protein stabilization but not STF reporter activation. It is possible that Wnt5a does not activate a transcriptional response when mFz4 is expressed alone in 293 cells due to the endogenous expression of mRor2. In addition, coreceptor expression and heterodimerization following ligand stimulation have previously been shown to enhance ligand binding and signal transduction in other systems [ 47, 48]. As Wnt5a-induced β-catenin stabilization in cells expressing mFz4 is less robust than Wnt3a-induced stabilization, overexpression of both mFz4 and LRP5 may be necessary for full potentiation of the canonical signal resulting in transcriptional activation. Furthermore, previous reports have suggested that the accumulation of β-catenin and Wnt signal transduction are separable events by showing that β-catenin protein levels alone do not dictate signal output but rather the phosphorylation state of β-catenin [ 49, 50]. Coexpression of LRP5 may be necessary for the dephosphorylation, and hence full activation, of β-catenin resulting in optimal signal transduction.

The disparities between previous reports and our current observations may be due to the fact that in this study, the effects of Wnt5a could be monitored immediately following Wnt5a protein addition as opposed to several hours or cell divisions following cellular expression, thereby allowing us to separate early and late effects of Wnt5a treatment. Activation of downstream transcriptional targets of Wnt5a, such as *Siah-2,* may contribute to the overall Wnt5a-mediated inhibition of Wnt/β-catenin signaling subsequent to the initial signaling events that we observed [ 16]. Additionally, in the proposed Wnt/Ca ^2+^ signaling model, Wnt5a-stimulated Ca ^2+^ flux leading to NLK activation is required for transcriptional inhibition [ 17]. However, Wnt1 has recently been shown to inhibit β-catenin–TCF–mediated transcription via activation of NLK, although Wnt1 has never been shown to induce Ca ^2+^ flux [ 33]. Thus, Wnt5a may exert its inhibitory effects through the activation of NLK in a similar Ca ^2+^-independent manner. Further experimentation into the mechanism by which Wnt5a inhibits canonical signaling is therefore necessary and will be addressed in subsequent reports.

**Figure 7:**
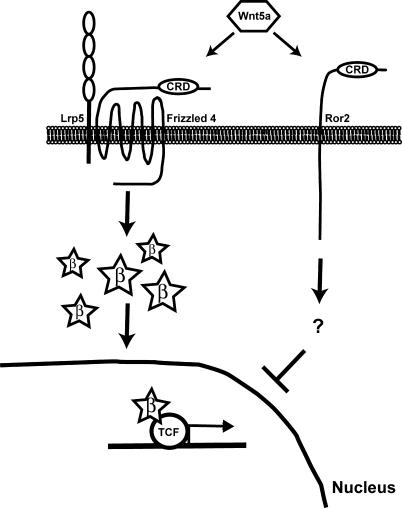
Model. Wnt5a Can Activate or Inhibit Canonical Wnt Signaling Depending on Receptor Context In the presence of Fz4 and LRP5, Wnt5a can activate β-catenin/TCF signaling. However, when Ror2 is expressed, Wnt5a inhibits canonical Wnt signaling downstream of β-catenin stabilization, at the level of TCF-mediated transcription.

In this study, we show that one Wnt ligand can function in two discreet pathways based on receptor availability ( Figure 7). Data in the literature suggest that this may also apply to other Wnts. For example, Wnt1 is known to activate canonical Wnt signaling through β-catenin and TCF and act as an oncogene [ 51]. However, using overexpression in various cell lines, Smit et al. presented evidence that Wnt1 inhibits TCF activity [ 33]. Another example is Wnt11, which has been implicated in the noncanonical convergence-extension pathway in zebrafish [ 52]. Recent work, by contrast, demonstrates that Wnt11 is the long-sought ligand that activates the β-catenin pathway in the early *Xenopus* embryo, showing that one Wnt can activate two different pathways, possibly also by activating different receptors [ 53].

Although the ability of one Wnt ligand to function in two distinct pathways based on receptor context is novel for the Wnt field, we note that in an entirely different system, opposing effects brought about by a single ligand have also been explained by the use of different receptor classes. This example is formed by the Netrins, ligands that can either attract or repel axons depending on whether they interact with the DCC (deleted in colorectal carcinoma) or UNC5 (UNCoordinated family member 5) families of receptors [ 54– 56]. Thus, ligands engaging multiple receptors to effect different signaling outcomes is not unprecedented in nature.

With as many as 19 mammalian homologs known, the question of whether all Wnt family members have evolved to signal in the same fashion is an important one. In this report, we address controversies in the literature regarding how one Wnt family member, Wnt5a, functions. Through quantitative kinetic analyses, we provide evidence for the first time that Wnt5a protein can directly inhibit canonical Wnt/β-catenin signaling and that the single-pass transmembrane receptor Ror2 is required to mediate this activity. Although Wnt5a can inhibit canonical Wnt signaling when Ror2 is expressed at detectable levels, Fz 4 and LRP5 coreceptor expression allows Wnt5a to signal in a canonical fashion. This ability of Wnt5a to toggle between two considerably diverse forms of signaling is particularly intriguing when one considers that Wnt5a has been classified as both a tumor suppressor and an oncogene in various cell types [ 57– 59]. While previously somewhat paradoxical, it is now clear that in the former role, Wnt5a expression may inhibit uncontrolled Wnt/β-catenin signaling in the presence of Ror2, whereas in the latter, Wnt5a could promote canonical Wnt signaling when Fz4 and LRP5 are expressed. Future study into specific receptor-ligand interactions will thus contribute to understanding the complexities of Wnt signaling.

## Materials and Methods

### Wnt5a purification

Wnt5a protein was purified from 6 L of media conditioned by mouse L cells stably overexpressing mouse *Wnt5a* (CRL-2814; American Type Culture Collection [ ATCC], Manassas, Virginia, United States) created in the laboratory as previously described [ 27] with the addition of a fourth purification step. Briefly, following a gel filtration sizing step, partially purified Wnt5a protein was bound to a copper-chelated resin and eluted with increasing concentrations of Imidazole (HiTrap Chelating HP; Amersham Biosciences, Little Chalfont, United Kingdom). Peak fractions were then further purified by heparin affinity chromatography as previously described. Typical yields of Wnt5a protein following final heparin affinity step range from 25 to 50 ng/μl as assessed by Coomassie blue staining.

### CRD protein purification

Serum-free media conditioned from 293 cells stably secreting CRD-IgG proteins was collected and bound to a Hitrap Protein A HP 1-ml column (Amersham Biosciences). Bound proteins were eluted with 100 mM citric acid (pH 3.0) and immediately made pH to neutral with 800 mM HEPES, 300 mM NaCl (pH 7.5) buffer supplemented with protease inhibitors. Protein from the peak fraction was incubated with protein A–Sepharose beads for 4 h at 4 °C and then washed 4 times with TNT buffer (see below). CRD-bound beads then incubated overnight at 4 °C with Wnt proteins. Beads were washed 4 times with TNT buffer, brought up in sample buffer, and analyzed for coimmunoprecipitation via Western blotting.

### cDNA constructs and antibodies

pcDNA3-mRor2 and mRor2ΔCRD constructs were obtained from the Minami lab [ 23]. We generated the mRor2 CRD-IgG construct by fusing the Fz8 signal sequence (bases 1 to 100) to the mRor2 CRD (bases 505 to 909 of the complementary DNA of mRor2) in frame with IgG as previously described [ 43]. The mRor2NT CRD-IgG construct corresponds to bases 1 to 909 of mRor2 fused to IgG. pRK5-mRor2-GPI construct was generated by fusing bases 1 to 1221 of mRor2 in frame to DAF-GPI. pRK5-DAF-GPI fusion protein vector was provided by the Nathans lab [ 43]. pEF1-mRor2-TM corresponds to bases 1 to 1303 of mRor2. Human LRP5 and LRP6 constructs in the CS2+ vector were obtained from the He lab [ 60]. pcDNA3.1- Fz 4 (FLAG-tagged) was provided by the Lefkowitz lab [ 15]. Fz 8 complementary DNA was subcloned into the pEF1-myc/his A vector (Invitrogen, Carlsbad, California, United States) and pEF1a-LacZ (Invitrogen) was used for β-galactosidase activity normalization. STF was obtained from the Moon lab [ 30]. All DNA segments generated by PCR were sequenced to rule out spurious mutations.

Antibodies were used at the following concentrations: rabbit anti-Wnt5A (1:1,000), rabbit anti-Fz4 (1:1,000), mouse 9E10 (1:200; Developmental Studies Hybridoma Bank at the University of Iowa, Iowa City, Iowa, United States), mouse anti-β-catenin (1:500; Santa Cruz Biotechnology, Santa Cruz, California, United States), mouse anti-GSK-3β (1:2,000; Transduction Labs, BD Biosciences Pharmingen, San Diego, California, United States), anti-phospho p44/p42 (1:1,000, Cell Signaling Technology, Beverly, Massachusetts, United States), and anti-total p44/p42 (1:1,000; Cell Signaling Technology). Proteins were detected using HRP-conjugated secondary antibodies (Santa Cruz Biotechnology) with ECL Western blot detection reagents (Amersham Biosciences). Anti-mouse Wnt5a antibody was generated by cloning a 167–base pair fragment corresponding to bases 757 to 924 of the complementary DNA of mWnt5a into the pGEX4T-1 vector. Purified mWnt5a-GST fusion protein was then injected into a rabbit following standard procedures (Josman Laboratories). The final bleed was subsequently affinity purified.

### Cell culture, mammalian cell transfection, and luciferase assays

HEK293 cells, mouse L cells, and their variants were cultured in DMEM, 10% FBS, and antibiotics.

To generate cell lines stably expressing Fz receptors, cells grown on 10-cm plates were transfected using LipofectAMINE2000 according to the manufacturer's instructions (Invitrogen). At 24 h post-transfection, cells were plated out in limiting dilution and cultured under neomycin selection (1 mg/ml G418) for 10 to 14 d. At least 20 single cell clones were screened for expression via Western blot analysis.

Transient transfections were performed with LipofectAMINE2000 (Invitrogen) on 293 cells, L cells, or stable cell line variants in six-well plates. Empty vector, CS2+-hLrp5, pcDNA3-mRor2, or derivatives were transfected (1 μg/well) together with the STF luciferase reporter (1 μg/well) and β-galactosidase (0.33 μg) (pEF-1α-LacZ; Invitrogen) plasmids. At 24 h post-transfection, cells were replated into a 96-well plate, allowed to recover for 6 to 8 h, and then treated in duplicate or triplicate with Wnt proteins as described in the text. Luciferase assays were performed using Dual-Light reporter gene assay system (Applied Biosystems). Relative luciferase units were measured and normalized against β-galactosidase activity at 48 h post-transfection. Error bars represent standard deviation, and each assay was performed at least two times.

### Cell extraction and Western blotting

For assaying Fz expression, cells were lysed in TNT buffer on ice (50 mM Tris-HCl, 150 mM NaCl, 1% Triton X-100). After clearing lysates with high-speed spin, protein supernatants were diluted into SDS sample buffer. In Erk1,2 phosphorylation assays, cells were lysed in hot sample buffer (62.5 mM Tris, 2% SDS, 10% glycerol, 50 mM DTT, 0.01% bromophenol blue) supplemented with protease inhibitors (Complete Inhibitors; Roche, Basel, Switzerland) and then passaged 10 times through a 25-gauge syringe. For visualization of β-Catenin accumulation in 293 cells, cells were lysed by hypotonic lysis: cells were scraped into hypotonic buffer (10 mM Tris-HCl, 0.2 mM MgCl _2_ [pH 7.4]) supplemented with protease and phosphatase inhibitors, incubated on ice for 10 min, and homogenized using a tight-fitting glass dounce. Sucrose and EDTA were added to final concentrations of 0.25 M and 1 mM, respectively. Lysates were then centrifuged at 20,000 × *g* for 1 h at 4 °C. Supernatant representing cytosolic cell fraction was then diluted into SDS sample buffer.

For immunoblotting, samples were resolved on SDS-10% polyacrylamide gels and transferred to nitrocellulose. The membranes were incubated for 1 h in blocking solution (1% BSA, 3% nonfat dry milk in Tris-buffered saline containing 0.2% Tween-20) and then overnight at 4 °C with antibody in blocking solution.

### Quantitative RT-PCR analysis

Qiagen RNeasy midi-kit (Valencia, California, United States) was used with on-column DNase step to prepare total RNA from each cell line. Then 1 μg of total RNA was converted to cDNA using random hexamer priming (Thermoscript RT-PCR System; Invitrogen). Quantitative real-time PCR analysis was performed in a Roche LightCycler 2.0 using LightCycler FastStart DNA Master ^PLUS^ SYBR Green I kit (Roche) with 0.5 μl of prepared cDNA and 0.5 μM forward and reverse primers per reaction. The primers for real-time PCR were designed using LightCycler Probe Design Software 2.0. Following 45 cycles of amplification, Ror2 expression in each cell line was normalized to HPRT expression following primer efficiency analysis. Reactions were performed in triplicate at least two times.

### Primers used

Primers used were human Ror2 F: 5′- ATGGAACTGTGTGACGTACCC-3′; mouse Ror2 F: 5′- TGGAACTGTGTGACGTACCC-3′; human/mouse Ror2 R: 5′- GCGAGGCCATCAGCTG-3′; human HPRT F: 5′- CAAGTTTGTTGTAGGATATGCCC-3′; human HPRT R: 3′- CGATGTCAATAGGACTCCAGA-3′; mouse HPRT F: 5′- TGTTGTTGGATATGCCCTTG-3′; and mouse HPRT R: 3′- TTGCGCTCATCTTAGGCTTT-3′.

### Immunofluorescence staining

For immunofluorescence microscopy, cells were grown on glass coverslips, fixed with 4% paraformaldehyde at 4 °C for 10 min, and permeabilized in methanol at 20 °C for 20 sec. The coverslips were then exposed to primary antibodies, followed by Cy3-conjugated secondary antibodies. Slides were mounted with Vectashield mounting media with Dapi (Vector Laboratories, Burlingame, California, United States) and fluorescence examined with a Zeiss Axioplan 2 microscope.

### Calcium flux assays

The 293 cells plated onto glass chamber slides (Nalge Nunc International, Rochester, New York, United States) were loaded with 1 μM fura-2 AM (Molecular Probes, Eugene, Oregon, United States) for 20 min prior to imaging. The cells were washed and then imaging was carried out on a previously described microscope system [ 61]. For time-lapse experiments, image pairs were collected at 340- and 380-nm excitation wavelength (510-nm emission) at 15-s intervals for 15 min following the addition of Wnt5a protein (500 ng/ml). Following Wnt protein incubation, cells were treated with ionomycin (1 μM) and immediately imaged for an additional 5 min. The ratio image, a pixel-by-pixel match of both excitation wavelengths, was calculated by computer software and the sequence of ratio images was processed. Microscope control, data acquisition, and image analysis were performed in MetaMorph (Universal Imaging, Molecular Dynamics, Sunnyvale, California, United States).

## Supporting Information

Figure S1 mRor2 Enhances Wnt5a-Mediated Inhibition of Canonical Wnt Signaling (A–C) Although Wnt5a protein shows inhibitory activity in 293 cells, it does not inhibit the STF reporter in 293Fz4 cells. Wnt5a-mediated inhibition is enhanced when mRor2 is stably expressed. At 24 h post-transfection, 293, 293Fz4, and 293Ror2 cells were treated with Wnt proteins for the indicated time points. Data are represented as relative light units (RLUs) ± SD rather than as average fold change.
(140 KB PDF)

## Abbreviations

APC: adenomatous polyposis coli gene product
CamKII: calcium/calmodulin-dependent kinase II
CRD: cysteine-rich Wnt binding domain
Dvl: Dishevelled
Fz: Frizzled
GSK-3β: glycogen synthase kinase-3β IgG
LEF: lymphoid enhancer factor
LPA: lysophosphatidic acid
LRP: low-density lipoprotein receptor-related protein
NLK: Nemo-like kinase
PKC: protein kinase C
PTX: pertussis toxin
STF: SuperTopFlash
TCF: T-cell factor
