# Supplementary material for: Purified Wnt5a Protein Activates or Inhibits β-Catenin–TCF Signaling Depending on Receptor Context
Source: PLoS Biol. 2006 Apr 4;4(4):e115. doi: 10.1371/journal.pbio.0040115 (PMC1420652; doi:10.1371/journal.pbio.0040115)

Figure S1

A.

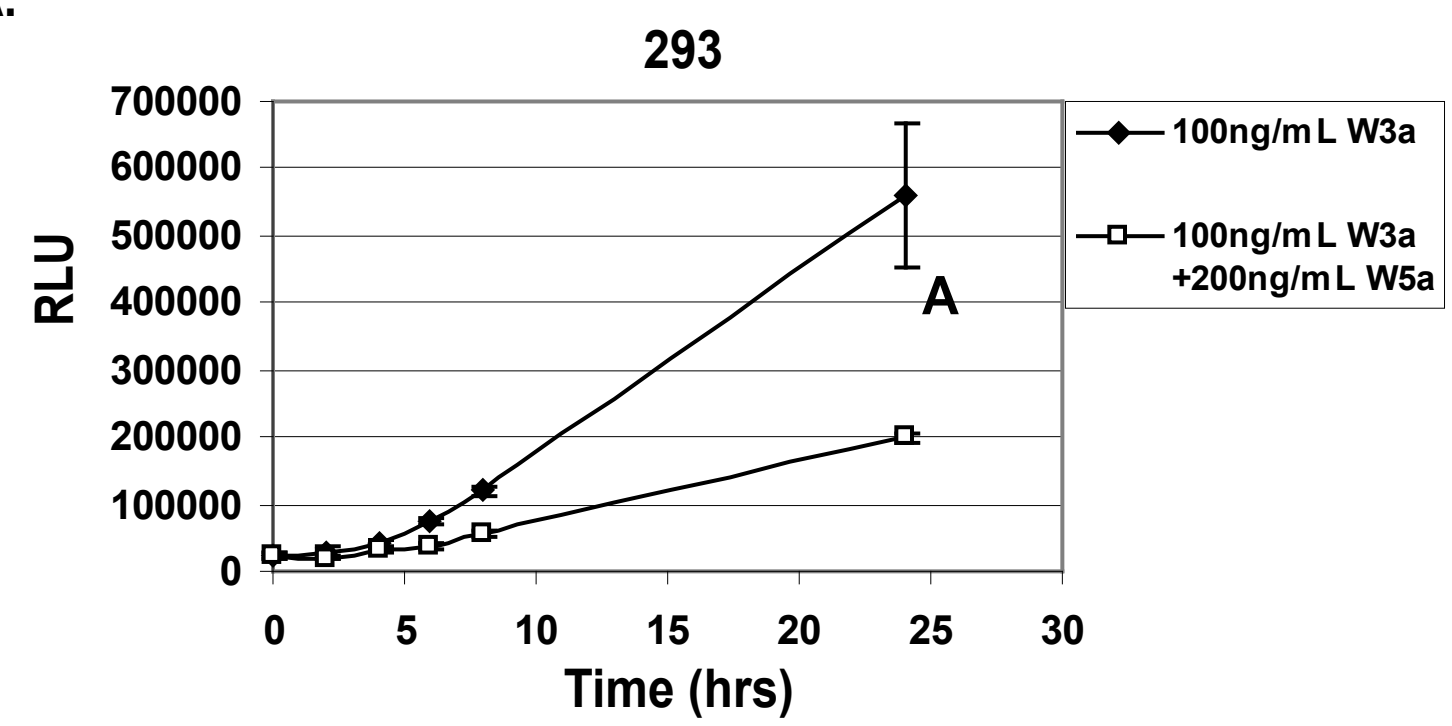

B.

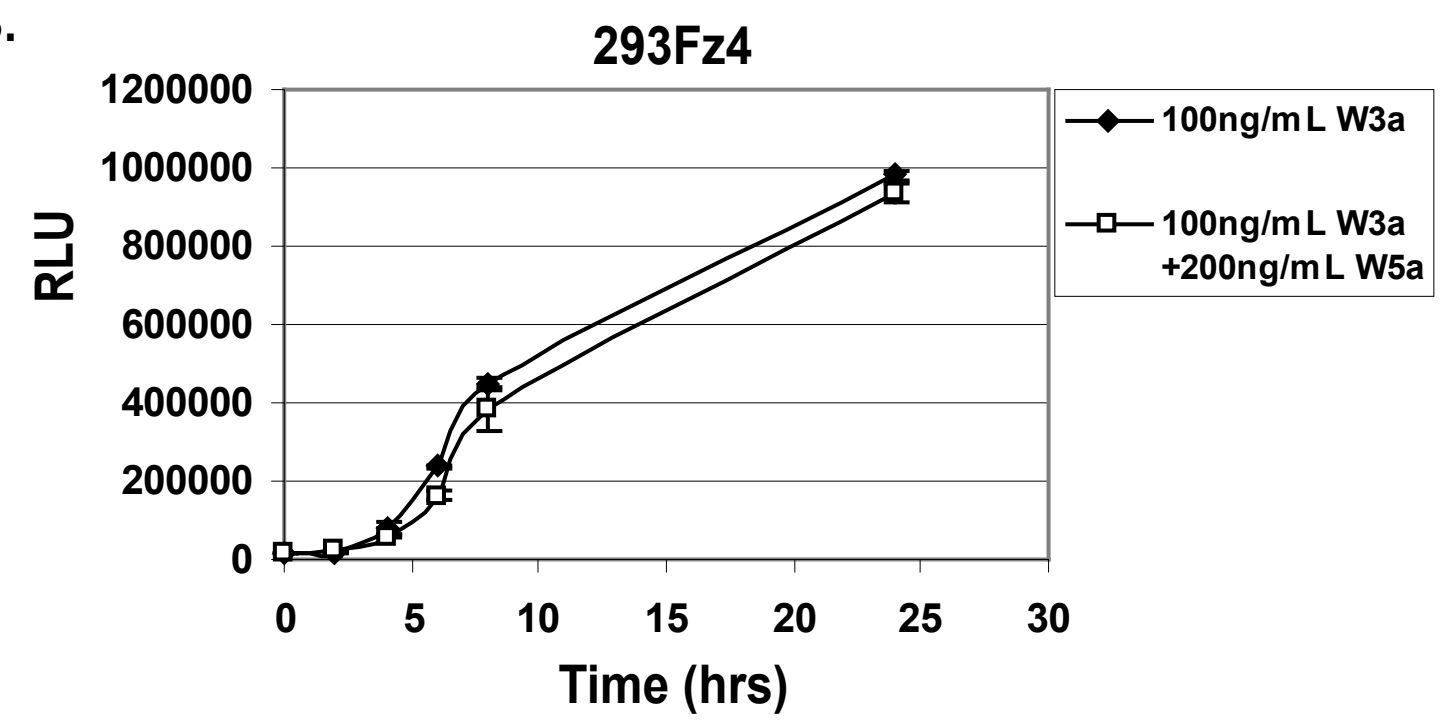

C.

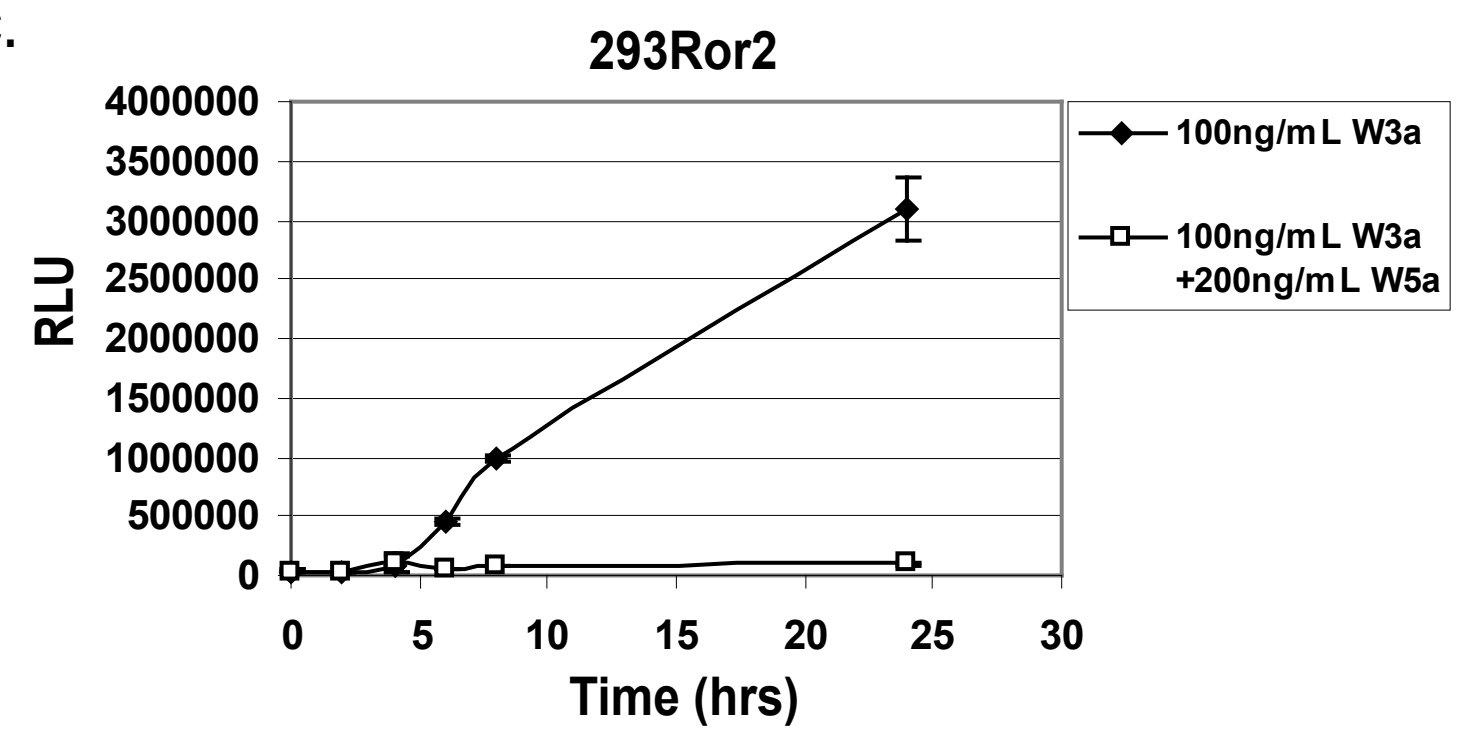

Supplement: Supplementary file 1 [file pbio.0040115.sg001.pdf]
